# Long-term quality of life of testicular cancer survivors differs according to applied adjuvant treatment and tumour type

**DOI:** 10.1007/s11764-024-01580-9

**Published:** 2024-04-24

**Authors:** Julia Heinzelbecker, Karla Kaßmann, Simone Ernst, Pia Meyer-Mabileau, Aleksandra Germanyuk, Miran Zangana, Gudrun Wagenpfeil, Carsten H. Ohlmann, Maximilian Cohausz, Michael Stöckle, Jan Lehmann

**Affiliations:** 1 Department of Urology and Pediatric Urology, Saarland University Medical Centre and Saarland University, Kirrberger Str. 100, 66421 Homburg/Saar, Germany; 2 Present Address: Department of Neurology, Heilig Geist-Krankenhaus, Graseggerstr. 105, 50737 Cologne-Longerich, Germany; 3 Present Address: Centre of Palliative Care and Pediatric Pain, Saarland University Medical Centre and Saarland University, Kirrbergerstr. 100, 66421 Homburg/Saar, Germany; 4 Institute of Medical Biometry, Epidemiology and Medical Informatics, Saarland University Campus Homburg/Saar, 66421 Homburg/Saar, Germany; 5 Present Address: Department of Urology, Johanniter Krankenhaus, Johanniterstr. 3-5, 53113 Bonn, Germany; 6 Present Address: Urologische Gemeinschaftspraxis Münster, Fürstenbergstr. 5, 48147 Münster, Germany; 7 Present Address: Urologische Gemeinschaftspraxis Pruener Gang, Pruener Grang 15, 24103 Kiel, Germany; 8 Present Address: Städtisches Krankenhaus Kiel, Chemnitzstr. 33, 24116 Kiel, Germany; 9 Present Address: Department of Urology and Pediatric Urology, University Medical Center Bonn (UKB), Bonn, Germany

**Keywords:** Testicular cancer, Survivor, Long-term, Quality of life, Therapy

## Abstract

**Purpose:**

To evaluate the quality of life (QoL) in long-term testicular cancer (TC) survivors.

**Methods:**

QoL was assessed in TC survivors treated between March 1976 and December 2004 (*n* = 625) using the EORTC-QLQ-C30 questionnaire, including a TC module. The assessment was performed at two time points (2006: response rate: *n* = 201/625 (32.2%), median follow-up (FU): 12.9 years (range 1.1–30.9); 2017: response rate: *n* = 95/201 (47.3%), median FU: 26.2 years (range: 13.0–41.2)). TC survivors were grouped according to treatment strategy, tumour entity, clinical stage and prognosis group. Linear and multiple linear regression analyses were performed, with age and time of follow-up as possible confounders.

**Results:**

Radiation therapy (RT) compared to retroperitoneal lymph node dissection (RPLND) was associated with a higher impairment of physical function (2017: *β* =  − 9.038; t(84) =  − 2.03; *p* = 0.045), role function (2017: *β* =  − 12.764; t(84) =  − 2.00; *p* = 0.048), emotional function (2006: *β* =  − 9.501; t(183) =  − 2.09; *p* = 0.038) and nausea (2006: *β* = 6.679; t(185) = 2.70; *p* = 0.008). However, RT was associated with a lower impairment of sexual enjoyment (2017: symptoms: *β* = 26.831; t(64) = 2.66; *p* = 0.010; functional: *β* = 22.983; t(65) = 2.36; *p* = 0.021). Chemotherapy (CT), compared to RPLND was associated with a higher impairment of role (2017: *β* =  − 16.944; t(84) =  − 2.62; *p* = 0.011) and social function (2017: β =  − 19.160; t(79) =  − 2.56; *p* = 0.012), more insomnia (2017: *β* = 19.595; t(84) = 2.25; *p* = 0.027) and greater concerns about infertility (2017: β = 19.830; t(80) = 2.30; *p* = 0.024). In terms of tumour type, nonseminomatous germ cell tumour (NSGCT) compared to seminoma survivors had significantly lower impairment of nausea (2006: *β* =  − 4.659; t(187) =  − 2.17; *p* = 0.031), appetite loss (2006: β =  − 7.554; t(188) =  − 2.77; *p* = 0.006) and future perspective (2006: *β* =  − 12.146; t(175) =  − 2.08; *p* = 0.039). On the other hand, surviving NSGCT was associated with higher impairment in terms of sexual problems (2006: *β* = 16.759; t(145) = 3.51; *p* < 0.001; 2017: *β* = 21.207; t(63) = 2.73; *p* = 0.008) and sexual enjoyment (2017: *β* =  − 24.224; t(66) =  − 2.76; *p* = 0.008).

**Conclusions:**

The applied adjuvant treatment and the tumour entity had a significant impact on the long-term QoL of TC survivors, even more than 25 years after the completion of therapy. Both RT and CT had a negative impact compared to survivors treated with RPLND, except for sexual concerns. NSGCT survivors had a lower impairment of QoL compared to seminoma survivors, except in terms of sexual concerns.

**Implications for Cancer Survivors:**

Implications for cancer survivors are to raise awareness of aspects of long-term and late effects on QoL in TC survivors; offer supportive care, such as psycho-oncological support or lifestyle modification, if a deterioration in QoL is noticed; and avoid toxic treatment without compromising a cure whenever possible.

**Supplementary Information:**

The online version contains supplementary material available at 10.1007/s11764-024-01580-9.

## Introduction

Testicular cancer (TC) is the most common solid malignancy in young men, with rising incidence rates [1]. As the disease has an excellent cure rate and affects mostly young men, TC survivors bear a significant risk of suffering from long-term consequences of therapy [2, 3]. There is a wide spectrum of adjuvant therapies that we can offer TC patients, including surveillance strategies, chemotherapy (CT) and retroperitoneal lymph node dissection (RPLND) [4]. Additionally, radiotherapy (RT) is in the armamentarium for TC treatment, and its use has continuously declined in recent decades due to severe concerns about long-term effects on morbidity and mortality [5]. Today, the majority of TC patients recover from acute side effects. However, it is the late adverse effects that have the greatest impact on long-term quality of life (QoL) and survival. Health-related QoL has become an important topic in clinical oncology in general, and especially in TC. Most cross-sectional studies have described a QoL in TC survivors comparable to that in the non-cancer population [6, 7]. However, little is known about how different therapeutic modalities impact the QoL of long-term TC survivors. The aim of our study was to evaluate health-related QoL in long-term survivors of TC, with a special emphasis on the applied adjuvant treatment strategies, tumour type, clinical stage and prognosis group.

## Patients and methods

### Study design and patients

**Fig. 1:**
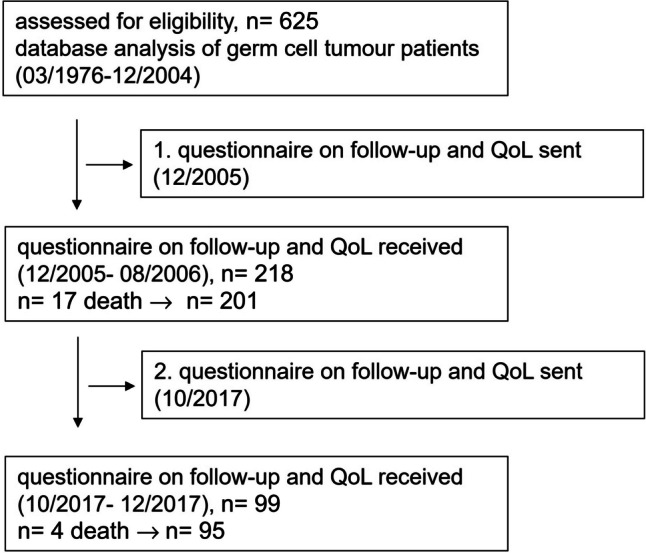
Flowchart

All patients with a diagnosis of testicular germ cell tumour treated between March 1976 and December 2004 at our department were identified through our database. In December 2005, a first standardized follow-up letter, and the EORTC-QLQ-C30 and TC module questionnaire were sent to these TC survivors, followed by a second one that was only sent to the respondents of the first one, in October 2017 (see Fig. 1, see results).

Clinicopathological patient data were collected from our patient database, a review of patient reports and answers to the survivors’ follow-up letters. Special attention was given to applied adjuvant treatment, tumour type, clinical stage according to Union International Contre le Cancer (UICC) and prognosis group according to International Germ Cell Cancer Cooperative Group (IGCCCG). The study was approved by the Ethics Committee, Saarbrücken, Germany.

### EORTC-QLQ-C30 and TC module

For the evaluation of QoL, the German version of the EORTC-QLQ-C30 (version 3.0) and the complementary TC module were used [8, 9]. The assessment of the questionnaires was performed according to the scoring manuals [8, 10]. The questionnaires consist of functional (i.e., emotional function) and symptom (i.e., nausea) scales. A higher score in the functional scales represents a better outcome, whereas a higher scale score in the symptom scales represents a worse outcome.

### Statistical analysis

Descriptive statistics are presented as the mean and standard deviation (SD), the median and range, or the number and percentage, as appropriate. Comparisons between the 2006 and 2017 questionnaires were performed with the *t* test for paired samples. We performed a linear regression and multiple linear regression analysis with age and length of follow-up as possible confounders. For the linear regression model, patients were grouped according to applied adjuvant therapy, tumour type, clinical stage and prognosis group according to IGCCCG. Regression results are presented as regression coefficients beta along with respective 95% confidence intervals or regression coefficients beta with test statistics *t*, degrees of freedom in parentheses and respective *p*-values. Primary RPLND was chosen as a reference group for the treatment modality as late effects that become manifest months to years after completion of treatment have not been described for RPLND, and because the surveillance group contained too few patients. We chose a significance cutoff of *p* < 0.05 in two-sided tests. All statistical analyses were conducted with the use of IBM SPSS software (version 28.0.1.0 (142)).

## Results

Six hundred and twenty-five TC patients who met the inclusion criteria were identified. In the 2006 questionnaire, we received 218 answers, of which 17 were sent by relatives of the patients to inform us of the patient’s death. Two hundred and one (32.2%) responders answered the questionnaire. For the 2017 questionnaire, we received 99 answers, 4 of which were relatives informing us of the death of the patient (see Fig. 1). Thus, 95 (47.3%) completed questionnaires remained for further analysis. The median age at diagnosis was 32 (range, 15–66; 2006) and 31 years (range, 15–53; 2017) and the median age at response of the questionnaire was 47 (range, 17.5–79.3; 2006) and 59 years (range, 29.4–81.8; 2017), respectively. The median follow-up since diagnosis was 12.9 years (range, 1.1–30.9; 2006) and 26.3 years (range, 13.0–41.5; 2017), respectively. The clinicopathological patient data are summarized in Table 1.

**Table 1:** Clinicopathological characteristics of the TC survivor collective. *n* = number of patients of which information was available; med. = median; y = year; CS = clinical stage; IGCCCG = International Germ Cell Cancer Cooperative Group; CT = chemotherapy; RPLND = retroperitoneal lymph node dissection; RT = radiation therapy; *including TC survivors who had chemotherapy and post-CT RPLND; ** including not applicable

	2006 (*n* = 625)		2017 (*n* = 201)	
	*n*		*n*	
Med. age at diagnosis (y, range)	201	32.0 (15.0–66.0)	95	31.0 (15.0–53.0)
med. age at questionnaire (y, range)	201	47.0 (17.5–79.3)	93	59.0 (29.4–81.8)
Med. follow-up (y, range)	201	12.9 (1.1–30.9)	93	26.3 (13.0–41.5)
Response rate		201 (32.2%)		95 (47.3%)
Tumour	201		95	
Seminoma		81 (40.3%)		36 (37.9%)
Non-seminoma		114 (56.7%)		56 (58.9%)
Burned out		1 (0.5%)		0 (0%)
Benign		4 (2.0%)		3 (3.2%)
Unknown		1 (0.5%)		0 (0%)
Clinical stage	201		95	
CS 1		124 (61.7%)		57 (60.0%)
CS > 1		68 (35.4%)		32 (33.7%)
Unknown**		9 (4.5%)		6 (6.3%)
Prognosis group (IGCCCG)	201		95	
None		124 (61.7%)		57 (60.0%)
Good		52 (25.9%)		25 (26.3%)
Intermediate		5 (2.5%)		2 (2.1%)
Poor		3 (1.5%)		0 (0%)
Unknown**		17 (8.5%)		11 (11.6)
Adjuvant therapy	201		95	
Surveillance		8 (4.0%)		3 (3.2%)
CT*		78 (38.8%)		38 (40.0%)
CT + RPLND		37 (47.4%)		18 (47.4%)
RT		61 (30.3%)		30 (31.6%)
RPLND (primary)		39 (19.4%)		17 (17.9%)
CT + RT		12 (6.0%)		5 (5.3%)
Unknown		3 (4.0%)		2 (2.1%)
Adjuvant therapy according to CS	201		95	
CS 1	123		57	
Surveillance		7 (5.7%)		2 (3.5%)
CT		22 (17.9%)		12 (21.1%)
RT		55 (44.7%)		26 (45.6%)
RPLND (primary)		36 (29.3%)		16 (28.1%)
CT + RT		3 (2.4%)		1 (1.8%)
CS > 1	68		32	
CT*		51 (75.0%)		24 (75.0%)
CT + RPLND		37 (72.5%)		18 (75.0%)
RT		5 (7.4%)		3 (9.4%)
RPLND (primary)		3 (4.4%)		1 (3.1%)
CT + RT		9 (13.2%)		4 (12.5%)
Unknown	10		6	
Relapse	201	19 (9.5%)	93	10 (10.8%)
Secondary malignancy	196	22 (11.2%)	89	16 (18.0%)
		Colorectal (3), skin (3), prostate (2), ureter (1), abdominal (1), lung (1), lymphoma (1), unknown (10)		Prostate (3), urinary bladder (2), colorectal (2), skin (2), cerebral (2), lung (1), kidney (1), unknown (3)
Arterial hypertension	184	52 (28.3%)	92	47 (51.1%)
Hyperlipidaemia	171	51 (29.8%)	91	32 (35.2%)
Fathered a child after diagnosis	194	22 (11.3%)	94	17 (18.1%)

Secondary malignancies occurred in 22 (11.2%, 2006) and 16 (18.0%, 2017) patients, with the most frequent being colorectal (*n* = 3), skin (*n* = 3) and prostate cancer (*n* = 2) in 2006; and prostate (*n* = 3), bladder (*n* = 2) colorectal (*n* = 2), skin (*n* = 2) and brain cancer (*n* = 2) in 2017. In 2006, 52 (28.3%) patients reported arterial hypertension, and in 2017 47 (51.1%). Hyperlipidaemia was reported in 51 (29.8%, 2006) and 32 (35.2%, 2017) patients, respectively; and 22 (11.3%, 2006) and 17 (18.1%, 2017) patients reported having fathered children after diagnosis (Table 1). The applied radiation dose ranged from 20 to 30 Gy; however, this was only known from 30 patients. The most often applied CT regimen was PEB, with two to four cycles being administered.

### QoL

The global QoL was 70.8 (± 20.6) in 2006 and 70.5 (± 21.4) in 2017. For the results of the different scales of the QLQ-C30 at the two different time points, see Suppl. 1. When comparing the different scores of the survivors who responded to both questionnaires, the mean global QoL (74.8 (± 18.7) vs. 70.5 (± 21.7), *p*-value = 0.048), physical function (93.0 (± 12.2) vs. 87.5 (± 18.6), *p*-value = 0.001) and role function (89.6 (± 18.9) vs. 83.3 (± 25.5), *p*-value = 0.010) were significantly lower in the responses from 2017 compared to 2006 (see Suppl. 2). In terms of symptom scales, patients in 2017 had significantly higher mean values of fatigue (24.8 (± 25.4) vs. 18.5 (± 21.9), *p*-value = 0.025) and dyspnoea (19.0 (± 28.6) vs. 13.2 (± 21.6), *p*-value = 0.048) (see Suppl. 2). Concerning TC-specific scales, the score for body image problems was significantly higher in 2017 (28.4 (± 36.5) vs. 19.5 (± 27.2), *p*-value = 0.016) (see Suppl. 2).

### QoL and adjuvant treatment modality

#### Univariate analysis in RT compared to RPLND survivors

**Fig. 2:**
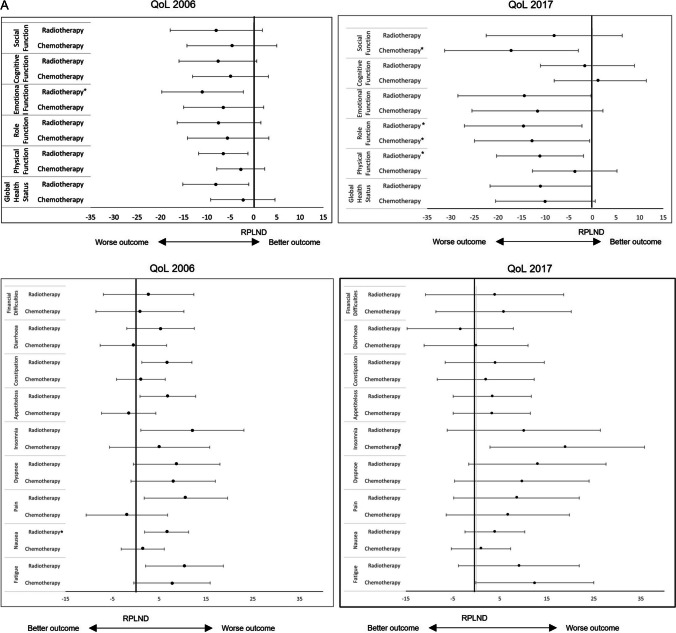

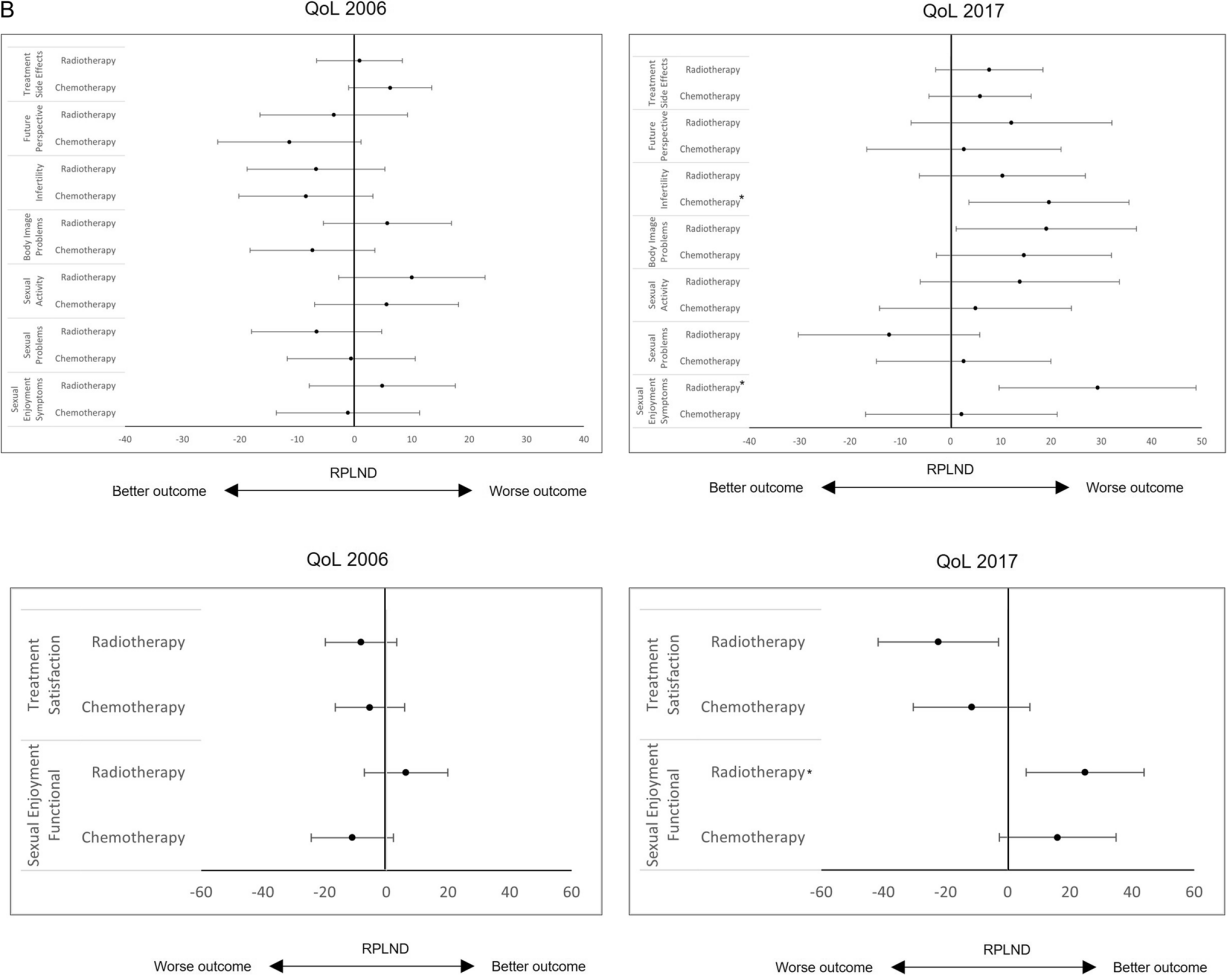
**A** Forest plots of the different scales of the QLQ-C30 questionnaire according to treatment modality. RPLND served as the reference treatment. For the functional scales (upper image border), higher scores represent a better outcome, whereas for the symptom scales (lower image border), higher scores represent a worse outcome. QoL, quality of life; RPLND, retroperitoneal lymph node dissection; *statistically significant *p* < 0.050 after multiple testing. **B** Forest plots of the different scales of the TC module questionnaire according to treatment modality. RPLND served as the reference treatment. For the symptom scales (upper image border), higher scores represent a worse outcome, whereas for the functional scales (lower image border), higher scores represent a better outcome. QoL, quality of life; RPLND, retroperitoneal lymph node dissection; *statistically significant *p* < 0.050 after multiple testing

Survivors receiving RT compared to RPLND had significantly poorer global health (2006: *p* = 0.025), including lower physical (2006: *p* = 0.017) and emotional function scores (2006: *p* = 0.014). They had significantly worse QoL concerning fatigue (2006: *p* = 0.014), nausea (2006: *p* = 0.006), pain (2006: *p* = 0.019), insomnia (2006: *p* = 0.031), appetite loss (2006: *p* = 0.025) and constipation (2006: *p* = 0.014). Some of these differences were still seen even with longer follow-up: a poorer global health status (2017, *p* = 0.047) as well as physical (2017: *p* = 0.019), role (2017: *p* = 0.022) and emotional function scores (2017: *p* = 0.046). RT was additionally associated with a higher impairment of body image problems (2017: *p* = 0.037). However, RT patients showed lower impairment in terms of sexual enjoyment (2017: symptoms: *p* = 0.004; functional: *p* = 0.011) (see Table 2 and Fig. 2A, B).

**Table 2:** Results of the QLQ-C30 and TC module according to applied adjuvant therapy. QoL = quality of life; *n* = number of patients; CI = confidence interval; RPLND = retroperitoneal lymph node dissection; CT = chemotherapy; RT = radiation therapy; ^a^Functional scale (low scores indicate high impairment or worse outcome); ^b^Symptom scale (high scores indicate high impairment or worse outcome); #reference group; *statistically significant p < 0.05; ^ꝉ^statistically trend p < 0.10; ^TC^Testicular cancer specific scales (TC module); multiple linear regression analysis with covariates: age AND length of follow-up

Parameters of QoL	Adjuvant Therapy	*n*	2006 Score β (95% CI)	Linear regression p-value	Multiple testing p-value	2017 Score β (95% CI)	n	Linear regression *p*-value	Multiple testing *p*-value
Global health status^a^	RPLND#	36	75.0 (69.0–81.1)			79.9 (70.6–89.1)	16		
	Surveillance	8	65.6 (44.0–87.2)	0.234		47.2 (12.5–81.9)	3	0.013*	0.010*
	CT	76	72.8 (59.8–85.7)	0.522		69.9 (50.1–89.7)	35	0.063^ꝉ^	
	RT	58	66.9 (53.8–80.1)	0.025*	0.068^ꝉ^	68.9 (48.9–89.0)	28	0.047*	0.068^ꝉ^
Physical function^a^	RPLND#	37	94.5 (90.0–99.0)			93.9 (86.0–101.8)	17		
	Surveillance	8	91.7 (75.4–107.9)	0.634		80.0 (49.9–110.1)	3	0.219	
	CT	78	91.8 (82.1–101.4)	0.294		90.2 (73.3–107.0)	37	0.422	0.062^ꝉ^
	RT	61	88.0 (78.2–97.8)	0.017*	0.170	82.8 (65.7–99.9)	30	0.019*	0.045*
Role function^a^	RPLND#	36	89.9 (82.1–97.5)			95.5 (84.8–106.2)	16		
	Surveillance	8	85.4 (57.8–113.0)	0.664		61.1 (20.3–101.9)	3	0.026*	0.021*
	CT	77	84.3 (67.9–100.8)	0.220		82.7 (59.8–105.6)	37	0.040*	0.011*
	RT	61	82.4 (65.7–99.1)	0.106		80.9 (57.7–104.0)	30	0.022*	0.048*
Emotional function^a^	RPLND#	36	80.2 (72.7–87.7)			85.1 (73.0–97.2)	16		
	Surveillance	8	67.7 (41.0–94.4)	0.201		55.6 (10.1–101.1)	3	0.082^ꝉ^	0.097^ꝉ^
	CT	76	73.7 (57.7–89.9)	0.138		73.5 (47.5–99.4)	35	0.097^ꝉ^	
	RT	60	69.2 (53.0–85.5)	0.014*	0.038*	70.7 (44.5–96.9)	28	0.046*	0.059^ꝉ^
Cognitive function^a^	RPLND#	36	87.4 (80.3–94.6)			83.2 (73.2–92.2)	16		
	Surveillance	8	81.3 (55.7–106.8)	0.507		83.3 (49.6–117.1)	3	0.990	
	CT	76	82.5 (67.2–97.8)	0.233		84.4 (65.1–103.6)	35	0.819	
	RT	60	79.8 (64.3–95.3)	0.071^ꝉ^		81.6 (62.2–101.1)	28	0.771	
Social function^a^	RPLND#	36	83.2 (74.8–91.7)			91.5 (79.1–103.9)	16		
	Surveillance	8	70.8 (40.7–101.0)	0.262		61.1 (14.6–107.6)	3	0.080^ꝉ^	0.074^ꝉ^
	CT	76	78.6 (60.5–96.7)	0.340		74.3 (47.8–100.9)	35	0.018*	0.012*
	RT	59	75.2 (56.9–93.6)	0.111		83.4 (56.6–110.2)	28	0.267	
Fatigue^b^	RPLND#	36	13.3 (6.2–20.4)			14.9 (3.8–26.0)	16		
	Surveillance	8	22.2 (− 3.2–47.6)	0.336		48.2 (6.5–89.8)	3	0.033*	0.038*
	CT	77	21.1 (5.8–36.3)	0.060^ꝉ^		27.3 (3.7–51.0)	36	0.052^ꝉ^	0.062^ꝉ^
	RT	60	23.7 (8.3–39.1)	0.014*	0.052^ꝉ^	24.0 (0.0–47.9)	29	0.163	
Nausea^b^	RPLND#	37	1.3 (− 2.7–5.4)			2.1 (− 3.4–7.6)	17		
	Surveillance	8	6.3 (− 8.2–20.7)	0.349		5.6 (− 15.4–26.5)	3	0.656	
	CT	78	2.8 (− 5.8–11.5)	0.519		3.1 (− 8.7–14.9)	37	0.747	
	RT	61	8.0 (− 0.8–16.7)	0.006*	0.008*	6.0 (− 5.8–17.9)	30	0.222	
Pain^b^	RPLND#	35	12.6 (4.9–20.2)			13.0 (1.4–24.6)	16		
	Surveillance	8	22.9 (− 4.2–50.1)	0.296		22.2 (− 21.2–65.6)	3	0.566	
	CT	76	10.7 (− 5.7–27.0)	0.669		19.7 (− 5.0–44.4)	36	0.314	
	RT	60	23.2 (6.7–39.8)	0.019*	0.073^ꝉ^	21.6 (− 3.4–46.5)	29	0.208	
Dyspnoea^b^	RPLND#	37	9.4 (1.5–17.2)			9.4 (− 3.1–21.9)	17		
	Surveillance	8	20.8 (− 7.5–49.1)	0.270		33.3 (− 14.4–81.1)	3	0.181	
	CT	78	17.4 (0.5–34.2)	0.082^ꝉ^		19.1 (− 7.7–45.9)	37	0.183	
	RT	60	18.1 (1.0–35.2)	0.064^ꝉ^		22.4 (− 4.7–49.5)	30	0.081^ꝉ^	
Insomnia^b^	RPLND#	37	16.3 (6.9–25.6)			14.8 (0.9–28.8)	17		
	Surveillance	8	33.3 (− 0.3–67.0)	0.168		33.3 (− 20.0–86.6)	3	0.353	
	CT	78	21.3 (1.3–41.4)	0.350		33.7 (3.8–64.6)	37	0.021*	0.027*
	RT	61	28.4 (8.0–48.7)	0.031*	0.162	24.9 (− 5.3–55.2)	30	0.223	
Appetite loss^b^	RPLND#	37	3.9 (− 1.2–9.0)			2.8 (− 4.3–9.9)	17		
	Surveillance	8	0.0 (− 18.3–18.3)	0.516		22.2 (− 5.0–49.4)	3	0.057	
	CT	78	2.4 (− 8.5–13.3)	0.616		6.1 (− 9.2–21.4)	36	0.424	
	RT	61	10.7 (− 0.3–21.8)	0.025*	0.056^ꝉ^	6.2 (− 9.2–21.6)	30	0.418	
Constipation^b^	RPLND#	36	2.5 (− 2.0–7.1)			5.8 (− 3.1–14.8)	16		
	Surveillance	8	0.0 (− 16.3–16.3)	0.672		11.1 (− 22.7–44.9)	3	0.670	
	CT	76	3.6 (− 6.1–13.4)	0.672		7.8 (− 11.4–27.1)	35	0.692	
	RT	59	9.2 (− 0.7–19.1)	0.014*	0.089^ꝉ^	9.8 (− 9.7–19.3)	28	0.449	
Diarrhoea^b^	RPLND#	36	9.3 (3.2–15.5)			10.5 (0.8–20.2)	16		
	Surveillance	8	12.5 (− 9.5–34.5)	0.695		11.1 (− 25.3–47.5)	3	0.965	
	CT	75	8.8 (− 4.4–22.1)	0.890		10.4 (− 10.3–31.2)	35	0.987	
	RT	59	14.6 (1.3–28.0)	0.149		7.1 (− 13.9–28.1)	28	0.547	
Financial difficulties^b^	RPLND#	36	14.3 (6.0–22.5)			10.4 (− 2.2–23.0)	16		
	Surveillance	8	29.2 (− 0.3–58.6)	0.168		16.7 (− 37.6–70.9)	2	0.766	
	CT	76	15.2 (− 2.5–32.8)	0.854		16.2 (− 10.8–43.2)	35	0.428	
	RT	60	17.0 (− 0.9–34.9)	0.581		14.3 (− 13.0–41.6)	28	0.604	
Treatment side effects^TC,b^	RPLND#	29	15.4 (9.2–21.7)			17.7 (8.8–26.5)	14		
	Surveillance	5	13.3 (− 10.9–37.6)	0.817		22.2 (− 14.0–58.4)	2	0.741	
	CT	56	21.7 (8.2–35.2)	0.091^ꝉ^		23.6 (4.5–42.6)	31	0.251	
	RT	41	16.3 (2.6–30.1)	0.816		25.4 (5.9–44.9)	20	0.152	
Future perspective^TC,b^	RPLND#	36	58.4 (47.6–69.2)			39.5 (22.7–56.3)	17		
	Surveillance	8	37.5 (− 0.7–75.7)	0.135		44.4 (− 19.9–108.8)	3	0.837	
	CT	73	47.0 (23.7–70.3)	0.075^ꝉ^		42.2 (6.1–78.4)	37	0.782	
	RT	56	54.7 (31.1–78.4)	0.581		51.7 (14.9–88.5)	27	0.229	
Infertility^TC,b^	RPLND#	36	31.3 (21.2–41.4)			6.9 (− 7.0–20.8)	17		
	Surveillance	8	16.7 (− 19.1–52.4)	0.260		33.3 (− 19.7–86.4)	3	0.183	
	CT	73	22.9 (1.1–44.7)	0.155		26.5 (− 3.3–56.4)	37	0.016*	0.024*
	RT	57	24.6 (2.5–46.8)	0.272		17.3 (− 13.2–47.7)	26	0.217	
Body image problems^TC,b^	RPLND#	35	24.7 (15.2–34.2)			14.0 (− 1.2–29.3)	17		
	Surveillance	8	33.3 (0.1–66.6)	0.475		77.8 (19.7–135.8)	3	0.004*	0.004*
	CT	73	17.4 (− 3.0–37.1)	0.186		28.7 (− 3.9–61.3)	37	0.098^ꝉ^	
	RT	57	30.4 (9.8–51.1)	0.312		33.1 (− 0.0–66.3)	28	0.037*	0.079^ꝉ^
Sexual activity^TC,b^	RPLND#	33	22.7 (11.9–33.5)			21.4 (4.9–37.9)	15		
	Surveillance	7	19.0 (− 20.0–58.1)	0.797		50.0 (− 9.6–109.6)	3	0.190	
	CT	63	28.3 (5.0–51.7)	0.380		26.4 (− 9.2–61.9)	34	0.604	
	RT	54	32.7 (9.2–56.3)	0.123		35.2 (− 1.1–71.5)	25	0.169	
Sexual problems^TC,b^	RPLND#	30	30.3 (20.6–39.9)			35.5 (20.4–50.5)	13		
	Surveillance	7	31.0 (− 2.7–64.6)	0.955		33.3 (− 19.1–85.7)	3	0.909	
	CT	57	29.7 (8.9–50.5)	0.920		38.1 (5.7–70.5)	29	0.761	
	RT	49	23.7 (2.7–44.6)	0.252		23.3 (− 9.8–56.4)	21	0.182	
Treatment satisfaction^TC,a^	RPLND#	36	22.7 (13.0–32.3)			38.7 (22.3–55.0)	17		
	Surveillance	8	29.2 (− 4.8–63.2)	0.600		11.1 (− 51.3–73.5)	3	0.237	
	CT	70	17.4 (− 3.5–38.3)	0.354		27.1 (− 8.0–62.3)	36	0.224	
	RT	56	14.6 (− 6.6–35.8)	0.168		16.4 (− 19.3–52.1)	27	0.025*	0.063^ꝉ^
Sexual enjoyment symptoms^TC,b^	RPLND#	31	19.2 (8.4–30.1)			12.7 (− 3.8–29.3)	13		
	Surveillance	7	33.3 (− 5.1–71.7)	0.314		44.4 (− 13.1–102.0)	3	0.128	
	CT	61	18.1 (− 5.2–41.4)	0.858		15.0 (− 10.7–50.6)	29	0.819	
	RT	50	24.1 (0.5–47.7)	0.453		42.1 (5.9–78.2)	23	0.004*	0.010*
Sexual enjoyment functional^TC,a^	RPLND#	30	31.0 (19.5–42.6)			10.8 (− 5.6–27.2)	13		
	Surveillance	7	19.0 (− 21.5–59.6)	0.415		44.4 (− 13.3–102.2)	3	0.109	
	CT	55	20.0 (− 4.9–44.9)	0.106		26.9 (− 8.3–62.1)	27	0.092^ꝉ^	
	RT	49	37.5 (12.4–62.5)	0.350		35.7 (0.4–71.1)	25	0.011*	0.021*

Values in bold indicates statistical significance at a *p*-value level of < 0.05

#### Multivariate regression analysis in RT compared to RPLND survivors

After correcting for age and length of follow-up as confounding parameters, RT compared to RPLND survivors in the 2006 questionnaire still had significantly worse emotional function scores (2006: *β* =  − 9.501; t(183) =  − 2.09; *p* = 0.038) and worse nausea symptoms (2006: *β* = 6.679; t(185) = 2.70; *p* = 0.008) and in the 2017 assessment worse physical (2017: *β* =  − 9.038; t(84) =  − 2.03; *p* = 0.045) and role function (2017: *β* =  − 12.764; t(84) =  − 2.00; *p* = 0.048). However, RT was associated with lower impairment in terms of sexual enjoyment (2017: symptom: *β* = 26.831; t(64) = 2.66; *p* = 0.010; functional: *β* = 22.983; t(65): 2.36; *p* = 0.021) (see Table 2 and Fig. 2A, B).

#### Univariate analysis in CT compared to RPLND survivors

With longer follow-up CT survivors had significantly lower role (2017: *p* = 0.040) and social function scores (2017: *p* = 0.018). Additionally, CT was associated with worse insomnia (2017: *p* = 0.021) and greater concerns about infertility (2017: *p* = 0.016) (see Table 2 and Fig. 2A, B).

#### Multivariate regression analysis in CT compared to RPLND survivors

All of these observations remained statistically significant after multiple testing with worse role function (2017: *β* =  − 16.944; t(84) =  − 2.62; *p* = 0.011) and social function scores (2017: *β* =  − 16.944; t(84) =  − 2.62; *p* = 0.011; 2017: *β* =  − 19.160; t(79) =  − 2.56; *p* = 0.012), worse insomnia (2017: *β* = 19.595; t(84) = 2.25; *p* = 0.027) and more concerns about infertility (2017: *β* = 19.830; t(80) = 2.30; *p* = 0.024) (see Table 2 and Fig. 2A, B) in CT survivors.

### QoL and tumour type

#### Univariate analysis of NSGCT compared to seminoma survivors

Compared to seminoma survivors, NSGCT survivors had a significantly better QoL concerning global health (2006: *p* = 0.005), including physical (2006: *p* = 0.004) and emotional function (2006: *p* = 0.047). Additionally, NSGCT survivors were less impaired by nausea (2006: *p* = 0.033), pain (2006: *p* = 0.012), dyspnoea (2006: *p* = 0.018) and appetite loss (2006: *p* = 0.001). They reported fewer body image problems (2006: *p* = 0.024) and concerns about the future (2006: *p* = 0.043). None of these differences were seen with longer follow-up in 2017. However, sexual problems (2006: *p* = 0.005; 2017: *p* = 0.019) and impairment of sexual enjoyment (2006: *p* = 0.047; 2017: *p* = 0.003) were worse at both follow-up time points. Treatment satisfaction was higher in NSGCT survivors (2017: *p* = 0.017) (see Suppl. 3 and Suppl. 6A and B).

#### Multivariate analysis of NSGCT compared to seminoma survivors

After correcting for age and length of follow-up as confounding parameters, NSGCT survivors still had less nausea and appetite loss (2006: *β* =  − 4.659; t(187) =  − 2.17; *p* = 0.031; β =  − 7.554; t(188) =  − 2.77; *p* = 0.006). Additionally, they were less concerned about the future (2006: *β* =  − 12.146; t(175) =  − 2.08; *p* = 0.039). Sexual problems (2006: *β* = 16.759; t(145) = 3.51; *p* < 0.001; 2017: *β* = 21.207; t(63) = 2.73; *p* = 0.008) and impairment of sexual enjoyment (2017: *β* =  − 24.224; t(66) =  − 2.76; *p* = 0.008) were more frequent (see Suppl. 3 and Suppl. 6A and B).

### QoL and clinical stage

There were hardly any differences in QoL between survivors according to clinical stage. Metastasized patients had fewer body image problems (2006: *p* = 0.019; β =  − 9.511; t(173) =  − 2.00; *p* = 0.047). However, they were more impaired by sexual problems (2006: *p* = 0.024; *β* = 15.002; t(143) = 3.09; *p* = 0.002) (see Suppl. 4).

### QoL and prognosis group

There were no noteworthy differences concerning the prognosis group in formerly metastasized survivors. Of note, the numbers of survivors with an intermediate and poor prognosis were very low (see Suppl. 5).

## Discussion

Many studies report on QoL in TC. However, reports on long-term QoL are sparse and there are only limited reports on the effects of applied adjuvant therapies [11–19]. Furthermore, many reports are lacking sufficient quality in reporting results, i.e., standardizing the assessment of QoL or testing multiple times. We hereby present a study with the so far longest follow-up in terms of QoL in TC survivors.

According to our study, all investigated adjuvant therapies for the treatment of TC impact long-term QoL even though different aspects of QoL are affected. Additionally, the tumour type, probably confounded by the difference in applied adjuvant therapies, impacts long-term QoL. The clinical stage and prognosis group had an only minor impact on long-term QoL.

### Effects of RT compared to RPLND on long-term QoL

After a median follow-up of 13 years, RT compared to RPLND was associated with impairment of emotional function and nausea, independently of age and length of follow-up. With a longer median follow-up of 26 years, there was an impairment in physical function. In the long run, RPLND compared to RT was associated with a higher impairment of sexual enjoyment.

Most studies on the emotional function of TC survivors report moderate to high levels of stress, depression and anxiety [20]. However, there only exist a few reports on the effects of the applied adjuvant treatments on later emotional well-being [11, 21, 22]. When comparing to other treatment modalities, Rudberg et al. reported that TC survivors treated with CT reported lower emotional well-being after a median follow-up of 8 years [11]. Fossa et al. reported similar results 3 years after therapy, in patients who were treated with both CT and RT [21]. Stuart et al. found a higher rate of depression in TC survivors who received RT [22]. According to our results, only TC survivors who received RT reported worse emotional well-being 13 years after treatment. All these studies differed in the length of follow-up. However, our study was the only one that corrected for age and length of follow-up.

Both CT and RT are well known to be associated with nausea as an acute side effect. However, the long-term and late outcome may also be affected. Fossa et al., already in 1988, reported a higher amount of nausea and vomiting in RT patients compared to those receiving surgery alone or CT 3 years after therapy [21]. We observed the same in our study, even after a median follow-up of 13 years regarding TC survivors. Of interest, Fossa et al. reported a raised death rate in germ cell tumour survivors due to benign gastrointestinal disorders [23]. Abdominal RT contributed considerably to this risk [23, 24]. They found out that gastroduodenal ulcers, as a long-term toxicity of RT, not only represent a significant morbidity but may also be a cause of death [23].

Interestingly, we found a reduction in physical function even 26 years after treatment in the RT group. It is well known that RT may lead to a reduction in physical function, mainly because the body is exposed to large amounts of cell debris and degradation products [25]. Nevertheless, it is astonishing that this phenomenon is detectable more than 25 years after the completion of treatment. However, late effects of treatment are generally considered irreversible and progressive over time [26].

We noticed a significantly worse outcome for RPLND compared to RT TC survivors in terms of sexual enjoyment 26 years after treatment. RPLND may be associated with andrological complications, the most important being retrograde ejaculation [27]. In terms of primary RPLND, the percentage of retrograde ejaculation ranges between 1 and 61% [27]. Erectile dysfunction after TC treatment seems to be a temporary impairment, as Capogrosso et al. reported a median time of erectile function recovery of 60, 60 and 70 months respectively for CT, RT and RPLND [28]. Aspects of sexual function, such as sexual activity or sexual problems, did not significantly differ between the adjuvant treatment modalities in our study. Nevertheless, it is likely that such problems may have an impact on sexual enjoyment and that this would affect primary RPLND more seriously than RT TC survivors. Rudberg et al. reported deteriorated sexual functioning in 14% of TC survivors after a mean follow-up of 8 years. Additionally, they found significantly lower sexual interest and a lower ability to enjoy sex in survivors who had undergone the treatment combination of RT plus CT and/or RPLND compared to other treatments [11]. Kim et al., after a median follow-up of 14 years, reported that CT increased the risk of sexual dysfunction in terms of delayed ejaculation, problem assessment and erectile dysfunction compared to non-cancer controls [29].

### Effects of CT compared to RPLND on long-term QoL

CT compared to RPLND, after a median follow-up of 26 years, was associated with a higher impairment of role and social function, insomnia and concerns about infertility.

We found role function, which is the ability to engage in work/household as well as leisure activities, to be significantly impaired in TC survivors receiving CT compared to RPLND. Van Leeuwen et al. reported on a significant minority of TC survivors who experienced work-related changes, especially affecting survivors with mental and physical health problems [30]. In contradiction to our own findings, Arai et al. reported a better working ability in survivors receiving CT and RT compared to those on surveillance at a median follow-up of 8 years. Moreover, survivors who underwent CT and RPLND reported the best working ability [31]. However, no standardized questionnaire was used. Kaasa et al. found no differences according to the treatment group at a mean of 5 years after therapy [32]. Ozen et al. reported a worse professional life for 17% of TC survivors when treated with RT and of 6% when treated with CT at a median follow-up of 5 years. However, a statistically significant effect of treatment modality was not observed [33].

In our study, social function in TC survivors treated with CT compared to RPLND was significantly impaired after a median follow-up of 26 years. Kim et al. reported the same observation when comparing to non-cancer controls after a median follow-up of 14 years, as assessed by the SF-36 questionnaire. However, in a multivariate analysis, their results could not be confirmed [14]. Vidrine et al. also reported significantly lower social function in patients treated with CT compared to surveillance at 3 and 12 months [13]. Nevertheless, in our evaluation, at only 13 years of follow-up, we found no such association. According to our results, CT treatment may contribute to a decreased ability to integrate and interact with others, even more than 20 years after treatment.

Additionally, survivors treated with CT compared to RPLND reported greater insomnia, even 26 years after treatment. There is some evidence that sleep disturbance negatively affects health and may be associated with increased mortality [34, 35]. Bumbasirevic et al. reported insomnia and financial difficulties as the most important effects that accounted for the worse QoL of TC survivors after a follow-up of 4 years [36]. Douchez et al., after 9 years of follow-up, found that about 30% of an NSGCT survivor collective reported insomnia [37]. Oechsle et al. also reported on sleep disturbances in 36% of TC survivors 12 years after therapy [15]. It is well known that some oncologic treatments may increase the risk of developing insomnia, either because of the emotional impact, the direct physiologic effects or the side effects [34]. Circulating plasma platinum is detectable in TC survivors even 20 years after the administration of CT [38], and there is evidence that cisplatin concentration correlates with the development of late effects including neurologic disturbances in TC survivors [39].

Interestingly, survivors who received CT were more concerned about infertility, even 26 years after treatment, compared to survivors who received RPLND. Yamashita et al. evaluated TC survivors with the QLQ-TC26 questionnaire and noticed a significant reduction in concerns about infertility in CT + RPLND survivors more than 10 years after treatment compared to less than 5 years after treatment [12]. In our survivor collective, we did not notice the same trend when a correlation analysis was performed (data not shown).

### Effects of tumour type on long-term QoL

NSGCT survivors had less nausea and appetite loss and were less concerned about the future compared to seminoma survivors after a median follow-up of 13 years. However, sexual problems and impairment of sexual enjoyment were more frequent, even at 26 years of follow-up.

Concerning tumour entity, Kim et al. found NSGCT survivors, compared to non-cancer controls, to be at higher risk in terms of sexual, erectile and ejaculatory dysfunction as well as problem assessment [29]. Jovanovski et al. reported on a possible increase in physical function among NSGCT survivors and a decrease in mental function over time since the cancer diagnosis [40]. Possible explanations for the differences in terms of tumour entity include different ages at diagnosis, with seminoma patients being on average older than NSGCT patients, and different applied adjuvant therapies. As we corrected for age and length of follow-up, age as an influencing factor should be of minor importance. Thus, the different treatment regimens applied should best explain our findings. We found NSGCT survivors to have less nausea and appetite loss compared to seminoma survivors, probably due to less RT being used. However, sexual problems and impairment of sexual enjoyment were more frequent in NSGCT survivors, corresponding to the higher amount of RPLND being used. Furthermore, NSGCT survivors were less concerned about the future.

The knowledge on the possible affection of long-term QoL in TC survivors stresses the importance of assessing their long-term QoL. This opens the door to offer supportive care, such as psycho-oncological support or lifestyle modifications. Additionally, it once again emphasizes the importance of a further reduction in treatment-related toxicity of TC patients without compromising cure, whenever possible.

Our study has several limitations. Survivors on surveillance are underrepresented in our study as surveillance regimens only gained importance in Germany after 2004. Thus, conclusions about adjuvant surveillance cannot be drawn from our study. In terms of adjuvant therapies, we did not discriminate between patients who had CT + RPLND (about 50% in each questionnaire group) and patients who only had CT as adjuvant treatment. We did not know the exact amount of radiation and CT applied in the RT and CT survivor collective. Primary RPLND served as the control group when comparing adjuvant treatment modalities. However, only patients in early stages of the disease receive primary RPLND. Nevertheless, in our analysis the impact of clinical stage or prognosis group on QoL was of only minor importance. There was a lack of assessment of sociodemographic parameters such as education, employment or family life as these questions were only implemented in the second questionnaire. We therefore decided not to report on them. The TC module we used as a supplement to the EORTC QLQ-C30 questionnaire was not validated during that time. Later, it was replaced by the validated EORTC QLQ-TC26 questionnaire. There was an essential change to one question: “Was sex less enjoyable for you compared to before your illness?” was changed to “To what extent was sex enjoyable to you?” [41]. The negative phrasing of the former question might have been misunderstood by some patients and thus might have provoked misleading answers.

## Conclusions

With the exception of sexual concerns, both RT and CT have a negative impact on the long-term QoL of TC survivors compared to primary RPLND. Compared to seminoma survivors, NSGCT survivors have better long-term QoL, except in terms of sexual concerns. Thus, both the applied adjuvant treatment and the tumour entity have a significant impact on the long-term QoL of TC survivors, even more than 25 years after the completion of therapy. Therefore, patient-reported outcomes of long-term TC survivors, as well as long-term toxicities, should be assessed and, whenever possible, actions undertaken to prevent or alleviate symptoms. Additionally, further efforts should be made towards reducing toxicity without compromising a cure in TC therapy.

## Supplementary Information

Below is the link to the electronic supplementary material.

Supplementary file1 (DOCX 28 KB)

Supplementary file2 (DOCX 49 KB)

Supplementary file3 (DOCX 14 KB)

Supplementary file4 (JPG 349 KB)

Supplementary file5 (JPG 306 KB)

## Data Availability

Available by request from the corresponding author.
