# Supplementary material for: Long-term quality of life of testicular cancer survivors differs according to applied adjuvant treatment and tumour type
Source: J Cancer Surviv. 2024 Apr 24;19(5):1651–65. doi: 10.1007/s11764-024-01580-9 (PMC12460406; doi:10.1007/s11764-024-01580-9)
Supplement: Supplementary file 1 — Supplementary file1 (DOCX 28 KB) [file 11764_2024_1580_MOESM1_ESM.docx]

|  | 2006 (n= 201) | | 2017 (n= 95) | |
| --- | --- | --- | --- | --- |
|  | n | Mean (±SD) | n | Mean (±SD) |
| **Global health status^a^** | 193 | 70.8 (±20.6) | 89 | 70.5 (±21.4) |
| **Functional scales^a^** | | | | |
| Physical function | 199 | 90.9 (±15.6) | 94 | 87.8 (±18.4) |
| Role function | 198 | 84.6 (±26.2) | 94 | 83.3 (±25.2) |
| Emotional function | 195 | 73.0 (±25.6) | 89 | 73.9 (±27.5) |
| Cognitive function | 195 | 82.3 (±24.2) | 89 | 83.1 (±19.7) |
| Social function | 194 | 77.9 (±28.6) | 89 | 80.0 (±28.2) |
| **Symptom scales^b^** | | | | |
| Fatigue | 196 | 20.8 (±24.3) | 91 | 24.7 (±25.3) |
| Nausea | 198 | 4.6 (±13.8) | 94 | 4.1 (±12.4) |
| Pain | 194 | 15.9 (±26.0) | 91 | 19.4 (±25.5) |
| Dyspnoea | 198 | 16.5 (±27.0) | 94 | 19.1 (±28.7) |
| Insomnia | 199 | 23.5 (±32.3) | 94 | 27.3 (±32.4) |
| Appetite loss | 199 | 5.5 (±17.7) | 93 | 6.1 (±16.3) |
| Constipation | 194 | 5.3 (±15.6) | 89 | 8.2 (±19.6) |
| Diarrhoea | 193 | 11.1 (±20.8) | 89 | 9.0 (±21.2) |
| Financial difficulties | 195 | 16.1 (±27.8) | 88 | 14.4 (±27.6) |
| **Testicular cancer specific scales (TC module)** | | | | |
| Treatment side effects^b^ | 143 | 18.3 (±19.1) | 71 | 23.3 (±18.2) |
| Treatment satisfaction^a^ | 182 | 17.8 (±31.9) | 90 | 24.8 (±38.6) |
| Future perspective^b^ | 186 | 51.1 (±36.4) | 91 | 45.8 (±38.4) |
| Infertility^b^ | 187 | 24.6 (±34.1) | 90 | 21.5 (±33.3) |
| Body image problems^b^ | 186 | 23.5 (±31.5) | 92 | 29.3 (±36.3) |
| Sexual activity^b^ | 170 | 28.8 (±34.9) | 82 | 29.7 (±34.6) |
| Sexual problems^b^ | 155 | 27.0 (±29.3) | 71 | 31.9 (±29.5) |
| Sexual enjoyment symptoms^b^ | 162 | 20.8 (±33.6) | 73 | 26.5 (±35.6) |
| Sexual enjoyment functional^a^ | 153 | 27.9 (±36.0) | 73 | 29.2 (±33.8) |

**Suppl. 1:** **Overview of the different scales of the QLQ-C30 an TC module.** n= number of patients in which the QLQ-C30 and TC module could be sufficiently evaluated; SD= standard deviation; ^a^low scores indicate high impairment or worse outcome; ^b^high scores indicate high impairment or worse outcome

|  | 2006 | | 2017 | |  |
| --- | --- | --- | --- | --- | --- |
|  | n | Mean (±SD) | n | Mean (±SD) | p-value |
| Global quality of life^a^ | 85 | 74.8 (±18.7) | 85 | 70.5 (±21.7) | **0.048*** |
| **Functional scales^a^** | | | | |  |
| Physical function | 91 | 93.0 (±12.2) | 91 | 87.5 (±18.6) | **0.001**** |
| Role function | 91 | 89.6 (±18.9) | 91 | 83.3 (±25.5) | **0.010*** |
| Emotional function | 85 | 74.4 (±26.0) | 85 | 73.9 (±27.5) | 0.866 |
| Cognitive function | 85 | 83.1 (±23.6) | 85 | 83.1 (±19.8) | 1.000 |
| Social function | 85 | 78.8 (±28.2) | 85 | 79.6 (±28.7) | 0.813 |
| **Symptom scales^b^** | | | | |  |
| Fatigue | 87 | 18.5 (±21.9) | 87 | 24.8 (±25.4) | **0.025*** |
| Nausea | 90 | 3.7 (±11.4) | 90 | 3.9 (±12.5) | 0.902 |
| Pain | 86 | 13.6 (±22.7) | 86 | 18.0 (±24.3) | 0.121 |
| Dyspnoea | 91 | 13.2 (±21.6) | 91 | 19.0 (±28.6) | **0.048*** |
| Insomnia | 91 | 20.5 (±30.5) | 91 | 26.7 (±32.3) | 0.075^ꝉ^ |
| Appetite loss | 90 | 4.8 (±17.0) | 90 | 6.3 (±16.5) | 0.374 |
| Constipation | 85 | 6.7 (±17.7) | 85 | 7.8 (±19.0) | 0.581 |
| Diarrhoea | 84 | 11.1 (±24.5) | 84 | 9.1 (±21.6) | 0.487 |
| Financial difficulties | 84 | 11.1 (±23.3) | 84 | 14.7 (±28.0) | 0.244 |
| **Testicular cancer specific scales (TC module)** | | | | |  |
| Treatment side effects^b^ | 57 | 22.7 (±23.0) | 57 | 23.0 (±19.0) | 0.891 |
| Treatment satisfaction^a^ | 82 | 18.3 (±33.6) | 82 | 24.8 (±38.1) | 0.145 |
| Future perspective^b^ | 86 | 44.6 (±35.6) | 86 | 44.6 (±39.1) | 1.000 |
| Infertility^b^ | 85 | 25.5 (±35.5) | 85 | 21.2 (±33.3) | 0.271 |
| Body image problems^b^ | 87 | 19.5 (±27.2) | 87 | 28.4 (±36.5) | **0.016*** |
| Sexual activity^b^ | 72 | 29.2 (±36.7) | 72 | 29.2 (±35.2) | 1.000 |
| Sexual problems^b^ | 61 | 28.7 (±29.0) | 61 | 29.8 (±29.5) | 0.772 |
| Sexual enjoyment symptoms^b^ | 64 | 21.9 (±36.7) | 64 | 25.5 (±35.0) | 0.366 |
| Sexual enjoyment functional^a^ | 62 | 29.0 (±37.4) | 62 | 30.1 (±34.5) | 0.845 |

**Suppl. 2:** **Comparison of the patient collective that responded to both questionnaires at the two different time points.** n= number of patients in which the QLQ-C30 and TC module could be sufficiently evaluated; SD= standard deviation; *= p-value <0.05, **= p-value <0.01; ^a^low scores indicate high impairment or worse outcome; ^b^high scores indicate high impairment or worse outcome
