# Supplementary material for: Long-term quality of life of testicular cancer survivors differs according to applied adjuvant treatment and tumour type
Source: J Cancer Surviv. 2024 Apr 24;19(5):1651–65. doi: 10.1007/s11764-024-01580-9 (PMC12460406; doi:10.1007/s11764-024-01580-9)
Supplement: Supplementary file 2 — Supplementary file2 (DOCX 49 KB) [file 11764_2024_1580_MOESM2_ESM.docx]

| Parameters of QoL | Tumour entity | n | 2006  Score β (95% CI) | Linear regression  p-value | Multiple testing  p-value | n | 2017  Score β (95% CI) | Linear regression  p-value | Multiple testing  p-value |
| --- | --- | --- | --- | --- | --- | --- | --- | --- | --- |
| Global health status^a^ | Seminoma#  Nonseminoma | 77  110 | 65.8 (61.4-70.3)  74.2 (63.9-84.6) | **0.005*** | 0.062^ꝉ^ | 34  52 | 69.7 (62.6-76.8)  70.5 (54.2-86.8) | 0.857 |  |
| Physical function^a^ | Seminoma#  Nonseminoma | 81  112 | 87.1 (83.8-90.5)  93.8 (85.9-101.3) | **0.004*** | 0.255 | 36  55 | 85.1 (79.2-91.0)  89.5 (75.8-103.1) | 0.263 |  |
| Role function^a^ | Seminoma#  Nonseminoma | 81  111 | 81.0 (75.4-86.5)  87.5 (74.5-100.5) | 0.081^ꝉ^ |  | 36  55 | 81.1 (73.0-89.3)  84.5 (65.8-103.3) | 0.525 |  |
| Emotional function^a^ | Seminoma#  Nonseminoma | 79  110 | 68.5 (63.0-74.0)  75.9 (63.1-88.8) | **0.047*** | 0.238 | 34  52 | 70.7 (61.5-79.7)  75.6 (54.6-96.7) | 0.402 |  |
| Cognitive function^a^ | Seminoma#  Nonseminoma | 79  110 | 80.5 (75.2-85.8)  83.3 (71.0-95.6) | 0.424 |  | 34  52 | 81.5 (74.9-88.0)  84.0 (68.9-99.1) | 0.563 |  |
| Social function^a^ | Seminoma#  Nonseminoma | 78  110 | 72.8 (66.6-79.1)  81.1 (66.6-95.5) | 0.050^ꝉ^ |  | 34  52 | 83.3 (74.0-92.7)  77.2 (55.7-98.8) | 0.324 |  |
| Fatigue^b^ | Seminoma#  Nonseminoma | 79  111 | 25.2 (19.9-30.5)  17.6 (5.4-29.9) | **0.033*** | 0.362 | 35  53 | 24.6 (16.3-32.9)  25.2 (6.0-44.3) | 0.922 |  |
| Nausea^b^ | Seminoma#  Nonseminoma | 81  111 | 6.9 (4.0-9.9)  2.7 (-4.1-9.5) | **0.033*** | **0.031*** | 36  55 | 6.6 (2.6-10.6)  2.4 (-6.7-11.6) | 0.114 |  |
| Pain^b^ | Seminoma#  Nonseminoma | 79  109 | 21.5 (15.9-27.2)  11.9 (-1.1-25.0) | **0.012*** | 0.069^ꝉ^ | 35  53 | 20.7 (12.3-29.1)  18.9 (-0.5-38.2) | 0.737 |  |
| Dyspnoea^b^ | Seminoma#  Nonseminoma | 80  112 | 21.7 (15.9-27.5)  12.5 (-0.9-25.9) | **0.018*** | 0.152 | 36  55 | 22.8 (13.5-32.1)  17.0 (-4.4-38.2) | 0.339 |  |
| Insomnia^b^ | Seminoma#  Nonseminoma | 81  112 | 27.8 (20.8-34.7)  20.8 (4.7-37.0) | 0.138 |  | 36  55 | 25.4 (14.9-35.9)  29.1 (4.9-53.2) | 0.597 |  |
| Appetite loss^b^ | Seminoma#  Nonseminoma | 81  112 | 10.3 (6.6-14.0)  2.1 (-6.6-10.8) | **0.001*** | **0.006*** | 36  54 | 7.0 (1.7-12.3)  5.6 (-6.6-17.7) | 0.675 |  |
| Constipation^b^ | Seminoma#  Nonseminoma | 78  110 | 7.0 (3.6-10.4)  4.2 (-3.7-12.2) | 0.231 |  | 34  52 | 11.1 (4.6-17.6)  6.4 (-8.6-21.4) | 0.274 |  |
| Diarrhoea^b^ | Seminoma#  Nonseminoma | 78  109 | 13.2 (8.6-17.7)  9.6 (-1.1-20.1) | 0.229 |  | 34  52 | 5.6 (-1.5-12.6)  11.5 (-4.6-27.2) | 0.196 |  |
| Financial difficulties^b^ | Seminoma#  Nonseminoma | 79  110 | 16.7 (10.6-22.8)  15.8 (1.6-29.9) | 0.824 |  | 34  51 | 11.1 (1.9-20.3)  17.0 (-4.2-38.2) | 0.332 |  |
| Treatment side effects^TC,b^ | Seminoma#  Nonseminoma | 59  81 | 18.8 (13.9-23.8)  18.2 (6.7-29.7) | 0.843 |  | 25  46 | 28.4 (21.3-35.6)  20.5 (4.4-36.6) | 0.081^ꝉ^ |  |
| Future perspective^TC,b^ | Seminoma#  Nonseminoma | 76  105 | 57.5 (49.3-65.6)  46.3 (27.4-65.3) | **0.043*** | **0.039*** | 33  55 | 51.5 (38.2-64.8)  41.8 (11.7-72.0) | 0.256 |  |
| Infertility^TC,b^ | Seminoma#  Nonseminoma | 78  104 | 21.8 (14.2-29.4)  27.0 (9.3-44.5) | 0.313 |  | 32  55 | 15.6 (4.2-27.1)  23.6 (-2.2-49.5) | 0.272 |  |
| Body image problems^TC,b^ | Seminoma#  Nonseminoma | 77  104 | 29.9 (22.9-36.9)  19.2 (3.0-35.5) | **0.024*** | 0.241 | 34  55 | 36.3 (23.9-48.1)  25.5 (-2.6-53.5) | 0.174 |  |
| Sexual activity^TC,b^ | Seminoma#  Nonseminoma | 73  93 | 32.9 (24.8-40.9)  26.0 (7.2-44.8) | 0.207 |  | 31  48 | 35.5 (23.1-47.9)  26.4 (-1.9-54.7) | 0.258 |  |
| Sexual problems^TC,b^ | Seminoma#  Nonseminoma | 69  81 | 20.0 (13.3-26.8)  33.3 (17.5-49.2) | **0.005*** | **<0.001*** | 25  43 | 21.3 (9.8-32.9)  38.8 (12.7-64.8) | **0.019*** | **0.008*** |
| Treatment satisfaction^TC,a^ | Seminoma#  Nonseminoma | 75  102 | 14.7 (7.5-21.9)  19.3 (1.6-36.0) | 0.339 |  | 33  54 | 12.1 (-0.7-25.0)  32.1 (2.9-61.3) | **0.017*** | 0.137 |
| Sexual enjoyment symptoms^TC,a^ | Seminoma#  Nonseminoma | 70  87 | 22.9 (15.0-30.8)  19.2 (0.6-37.7) | 0.492 |  | 27  43 | 35.8 (22.6-49.0)  20.2 (-9.9-50.2) | 0.068^ꝉ^ |  |
| Sexual enjoyment functional^TC,a^ | Seminoma#  Nonseminoma | 68  80 | 34.8 (26.2-43.4)  22.9 (2.6-43.3) | **0.047*** | 0.444 | 31  40 | 43.1 (31.5-54.5)  19.2 (-7.7-46.0) | **0.003*** | **0.008*** |

**Suppl. 3: Results of the QLQ-C30 and TC module according to tumour entity.** QoL= quality of life; n= number of patients; CI= confidence interval; ^a^Functional scale (low scores indicate high impairment or worse outcome); ^b^Symptom scale (high scores indicate high impairment or worse outcome); #reference group; *statistically significant p <0.05; ^ꝉ^statistically trend p <0.10; ^TC^Testicular cancer specific scales (TC module); multiple linear regression analysis with covariates: age AND length of follow-up.

| Parameters of QoL | CS | n | 2006  Score β (95% CI) | Linear regression  p-value | Multiple testing  p-value | n | 2017  Score β (95% CI) | Linear regression  p-value | Multiple testing  p-value |
| --- | --- | --- | --- | --- | --- | --- | --- | --- | --- |
| Global health status^a^ | CS 1#  CS> 1 | 118  66 | 70.9 (67.1-74.4)  70.1 (60.0-80.2) | 0.796 |  | 54  29 | 71.9 (66.1-77.7)  66.4 (50.7-82.1) | 0.267 |  |
| Physical function^a^ | CS 1#  CS> 1 | 123  67 | 91.2 (88.5-93.9)  90.5 (83.2-97.9) | 0.773 |  | 57  31 | 87.4 (82.4-92.3)  86.7 (73.3-100.0) | 0.868 |  |
| Role function^a^ | CS 1#  CS> 1 | 123  66 | 85.1 (80.6-89.6)  85.6 (73.4-97.8) | 0.896 |  | 57  31 | 83.0 (76.3-89.9)  80.6 (62.4-98.8) | 0.678 |  |
| Emotional function^a^ | CS 1#  CS> 1 | 120  66 | 73.3 (68.7-77.9)  72.2 (60.0-84.5) | 0.790 |  | 54  29 | 73.0 (65.4-80.5)  71.3 (50.9-91.6) | 0.788 |  |
| Cognitive function^a^ | CS 1#  CS> 1 | 120  66 | 82.6 (78.3-87.0)  81.1 (69.4-92.7) | 0.670 |  | 54  29 | 80.9 (75.5-86.3)  85.6 (71.1-100.2) | 0.303 |  |
| Social function^a^ | CS 1#  CS> 1 | 119  66 | 77.7 (72.6-82.8)  79.0 (65.4-92.7) | 0.762 |  | 54  29 | 80.6 (73.1-88.0)  78.2 (58.1-98.3) | 0.707 |  |
| Fatigue^b^ | CS 1#  CS> 1 | 121  66 | 20.2 (15.9-24.5)  22.1 (10.5-33.6) | 0.615 |  | 54  31 | 25.9 (19.0-32.9)  26.2 (7.7-44.7) | 0.967 |  |
| Nausea^b^ | CS 1#  CS> 1 | 122  67 | 5.5 (2.9-8.0)  3.5 (-3.3-10.2) | 0.357 |  | 57  31 | 2.9 (-0.4-6.3)  7.0 (-2.0-16.0) | 0.155 |  |
| Pain^b^ | CS 1#  CS> 1 | 121  64 | 18.6 (13.9-23.3)  11.5 (-1.3-24.2) | 0.082^ꝉ^ |  | 54  31 | 17.6 (10.6-24.6)  25.8 (7.3-44.3) | 0.161 |  |
| Dyspnoea^b^ | CS 1#  CS> 1 | 122  67 | 15.3 (10.6-20.0)  16.9 (4.3-29.5) | 0.688 |  | 57  31 | 17.0 (9.3-24.6)  26.9 (6.4-47.4) | 0.129 |  |
| Insomnia^b^ | CS 1#  CS> 1 | 123  67 | 25.2 (19.5-30.9)  20.9 (5.6-36.2) | 0.378 |  | 57  31 | 24.0 (15.5-32.5)  36.6 (13.8-59.3) | 0.083^ꝉ^ |  |
| Appetite loss^b^ | CS 1#  CS> 1 | 123  67 | 5.4 (2.4-8.4)  5.0 (-3.0-13.0) | 0.861 |  | 57  30 | 4.1 (-0.3-8.4)  11.1 (-0.6-22.8) | 0.063^ꝉ^ |  |
| Constipation^b^ | CS 1#  CS> 1 | 119  66 | 5.9 (3.2-8.6)  3.5 (-3.8-10.8) | 0.313 |  | 54  29 | 8.6 (3.7-13.5)  4.6 (-8.6-17.8) | 0.334 |  |
| Diarrhoea^b^ | CS 1#  CS> 1 | 119  65 | 12.6 (8.8-16.4)  8.7 (-1.5-18.9) | 0.233 |  | 54  29 | 9.9 (3.9-15.8)  9.2 (-6.8-25.2) | 0.893 |  |
| Financial difficulties^b^ | CS 1#  CS> 1 | 120  66 | 15.8 (10.8-20.8)  15.2 (1.7-28.6) | 0.873 |  | 53  29 | 13.8 (6.1-21.6)  18.4 (-2.4-39.2) | 0.489 |  |
| Treatment side effects^TC,b^ | CS 1#  CS> 1 | 82  55 | 16.2 (12.1-20.3)  21.2 (10.5-31.9) | 0.131 |  | 42  26 | 23.3 (17.6-29.0)  25.0 (10.1-39.9) | 0.711 |  |
| Future perspective^TC,b^ | CS 1#  CS> 1 | 114  64 | 54.7 (48.0-61.3)  45.3 (27.6-63.1) | 0.097 ^ꝉ^ |  | 55  30 | 47.3 (37.0-57.6)  46.7 (19.0-74.4) | 0.945 |  |
| Infertility^TC,b^ | CS 1#  CS> 1 | 116  63 | 24.4 (18.2-30.6)  24.3 (7.7-40.9) | 0.987 |  | 53  31 | 15.1 (6.5-23.7)  29.0 (6.4-51.7) | 0.053 ^ꝉ^ |  |
| Body image problems^TC,b^ | CS 1#  CS> 1 | 115  63 | 27.2 (21.6-32.9)  15.9 (0.7-31.0) | **0.019*** | **0.047*** | 55  31 | 27.9 (18.2-37.6)  32.2 (6.5-58.1) | 0.590 |  |
| Sexual activity^TC,b^ | CS 1#  CS> 1 | 107  56 | 28.0 (21.4-34.6)  28.3 (10.4-46.1) | 0.967 |  | 50  26 | 30.7 (20.8-40.6)  32.1 (5.2-58.9) | 0.871 |  |
| Sexual problems^TC,b^ | CS 1#  CS> 1 | 99  49 | 23.7 (17.9-29.5)  35.4 (19.5-51.2) | **0.024*** | **0.002*** | 43  22 | 31.0 (21.8-40.2)  34.1 (9.1-59.1) | 0.698 |  |
| Treatment satisfaction^TC,a^ | CS 1#  CS> 1 | 113  61 | 17.4 (11.6-23.2)  15.8 (0.3-31.4) | 0.753 |  | 54  31 | 26.5 (16.3-36.8)  18.3 (-8.9-45.4) | 0.334 |  |
| Sexual enjoyment symptoms^TC,a^ | CS 1#  CS> 1 | 101  53 | 21.5 (14.9-28.0)  18.2 (0.6-35.9) | 0.568 |  | 45  23 | 31.9 (21.5-42.2)  17.4 (-10.7-45.5) | 0.109 |  |
| Sexual enjoyment functional^TC,a^ | CS 1#  CS> 1 | 97  49 | 30.6 (23.3-37.9)  24.5 (4.6-44.4) | 0.340 |  | 45  24 | 30.4 (20.1-40.7)  29.2 (1.4-56.9) | 0.891 |  |

**Suppl. 4: Results of the QLQ-C30 and TC module according to clinical stage.** QoL= quality of life; CS= clinical stage; n= number of patients; CI= confidence interval; ^a^Functional scale (low scores indicate high impairment or worse outcome); ^b^Symptom scale (high scores indicate high impairment or worse outcome); #reference group; *statistically significant p <0.05; ^ꝉ^statistically trend p <0.10; ^TC^Testicular cancer specific scales (TC module); multiple linear regression analysis with covariates: age AND length of follow-up.

| Parameters of QoL | Prognosis group | n | 2006  Score β (95% CI) | Linear regression  p-value | Multiple testing  p-value | n | 2017  Score β (95% CI) | Linear regression  p-value | Multiple testing  p-value |
| --- | --- | --- | --- | --- | --- | --- | --- | --- | --- |
| Global health status^a^ | good#  > good | 51  7 | 68.8 (62.4-75.1)  75.0 (50.4-99.6) | 0.499 |  | 23  1 | 65.9 (55.7-76.2)  33.3 (-27.1-93.8) | 0.192 |  |
| Physical function^a^ | good#  > good | 51  8 | 88.6 (84.3-93.0)  98.3 (82.1-114.5) | 0.106 |  | 24  2 | 85.8 (76.1-95.5)  83.3 (38.6-128.1) | 0.884 |  |
| Role function^a^ | good#  > good | 51  7 | 83.9 (75.9-90.1)  97.6 (70.0-125.2) | 0.159 |  | 24  2 | 79.9 (67.3-92.4)  75.0 (17.3-132.7) | 0.826 |  |
| Emotional function^a^ | good#  > good | 51  7 | 69.6 (62.4-76.9)  83.3 (55.2-111.4) | 0.193 |  | 23  1 | 72.5 (61.5-83.4)  8.3 (-56.2-72.9) | **0.021*** | **0.035*** |
| Cognitive function^a^ | good#  > good | 51  7 | 78.4 (72.0-84.9)  92.9 (67.9-117.8) | 0.125 |  | 23  1 | 87.7 (80.7-94.6)  83.3 (42.4-124.3) | 0.793 |  |
| Social function^a^ | good#  > good | 51  7 | 75.5 (67.5-83.5)  92.9 (62.0-123.7) | 0.135 |  | 23  1 | 80.4 (67.8-93.0)  66.7 (-7.7-141.0) | 0.648 |  |
| fatigue^b^ | good#  > good | 51  7 | 23.3 (16.0-30.6)  17.5 (-10.9-45.8) | 0.580 |  | 24  2 | 24.5 (12.7-36.3)  50.0 (-4.3-104.3) | 0.229 |  |
| Nausea^b^ | good#  > good | 51  8 | 3.6 (0.8-6.4)  0.0 (-10.4-10.4) | 0.350 |  | 24  2 | 6.9 (-1.9-15.8)  8.3 (-32.5-49.2) | 0.929 |  |
| Pain^b^ | good#  > good | 50  6 | 13.0 (7.0-19.0)  2.8 (-21.6-27.2) | 0.269 |  | 24  2 | 26.4 (12.5-40.3)  50.0 (-13.9-113.9) | 0.340 |  |
| Dyspnoea^b^ | good#  > good | 51  8 | 19.0 (10.5-27.4)  16.7 (-14.7-48.0) | 0.842 |  | 24  2 | 26.4 (12.8-40.0)  50.0 (-13.9-113.9) | 0.330 |  |
| Insomnia^b^ | good#  > good | 51  8 | 22.9 (14.2-31.5)  12.5 (-19.7-44.7) | 0.380 |  | 24  2 | 33.3 (18.6-48.1)  83.3 (15.4-151.3) | 0.064 ^ꝉ^ |  |
| Appetite loss^b^ | good#  > good | 51  8 | 5.9 (1.3-10.4)  0.0 (-16.8-16.8) | 0.343 |  | 24  1 | 11.1 (0.4-21.8)  33.3 (-31.0-97.6) | 0.400 |  |
| Constipation^b^ | good#  > good | 51  7 | 3.3 (-0.4-6.9)  0.0 (-14.1-14.1) | 0.535 |  | 23  1 | 2.9 (-1.3-7.1)  0.0 (-24.5-24.5) | 0.770 |  |
| Diarrhoea^b^ | good#  > good | 50  7 | 10.0 (4.5-15.5)  0.0 (-21.1-21.1) | 0.205 |  | 23  1 | 8.7 (-1.2-18.6)  0.0 (-58.6-58.6) | 0.714 |  |
| Financial difficulties^b^ | good#  > good | 51  7 | 17.9 (8.1-25.9)  14.3 (-20.1-48.7) | 0.833 |  | 23  1 | 18.8 (3.3-34.4)  33.3 (-58.5-125.1) | 0.697 |  |
| Treatment side effects^TC,b^ | good#  > good | 42  7 | 22.9 (16.4-29.4)  13.5 (-10.1-37.1) | 0.276 |  | 20  2 | 24.7 (15.0-34.5)  38.9 (-3.2-81.0) | 0.372 |  |
| Future perspective^TC,b^ | good#  > good | 49  7 | 47.6 (36.3-58.9)  42.9 (-0.3-86.1) | 0.766 |  | 23  2 | 44.9 (27.6-62.2)  100.0 (21.6-178.4) | 0.075 ^ꝉ^ |  |
| Infertility^TC,b^ | good#  > good | 48  7 | 27.1 (16.3-37.9)  28.6 (-12.4-69.6) | 0.922 |  | 24  2 | 30.6 (13.9-47.2)  66.7 (-10.1-143.5) | 0.227 |  |
| Body image problems^TC,b^ | good#  > good | 48  7 | 18.8 (10.9-26.6)  9.5 (-20.4-39.5) | 0.405 |  | 24  2 | 30.6 (15.4-45.7)  66.7 (-3.0-136.3) | 0.184 |  |
| Sexual activity^TC,b^ | good#  > good | 44  6 | 30.3 (19.1-41.5)  30.6 (-13.1-74.2) | 0.988 |  | 20  2 | 29.2 (11.0-47.4)  50.0 (-28.6-128.6) | 0.480 |  |
| Sexual problems^TC,b^ | good#  > good | 39  4 | 39.7 (29.7-49.7)  33.3 (-9.5-76.1) | 0.695 |  | 17  2 | 33.3 (19.9-46.7)  83.3 (28.6-138.1) | **0.021*** | **0.009*** |
| Treatment satisfaction^TC,a^ | good#  > good | 47  7 | 18.4 (9.0-27.9)  4.8 (-30.8-40.3) | 0.299 |  | 24  2 | 16.7 (2.5-30.9)  16.7 (-48.7-82.0) | 1.000 |  |
| Sexual enjoyment symptoms^TC,a^ | good#  > good | 41  5 | 20.3 (10.3-30.4)  6.7 (-34.0-47.3) | 0.372 |  | 18  2 | 13.0 (-0.9-26.9)  50.0 (-7.9-107.9) | 0.094 ^ꝉ^ |  |
| Sexual enjoyment functional^TC,a^ | good#  > good | 39  4 | 24.8 (14.4-35.2)  8.3 (-36.2-52.8) | 0.336 |  | 19  2 | 29.8 (12.7-47.0)  0.0 (-72.7-72.7) | 0.275 |  |

**Suppl. 5: Results of the QLQ-C30 and TC module according to prognosis group.** QoL= quality of life; n= number of patients; CI= confidence interval; good= good prognosis group according to IGCCCG; >good= intermediate and poor prognosis group according to IGCCCG; ^a^Functional scale (low scores indicate high impairment or worse outcome); ^b^Symptom scale (high scores indicate high impairment or worse outcome); #reference group; *statistically significant p <0.05; ^ꝉ^statistically trend p <0.10; ^TC^Testicular cancer specific scales (TC module); multiple linear regression analysis with covariates: age AND length of follow-up.
