# Supplementary material for: Long-term quality of life of testicular cancer survivors differs according to applied adjuvant treatment and tumour type
Source: J Cancer Surviv. 2024 Apr 24;19(5):1651–65. doi: 10.1007/s11764-024-01580-9 (PMC12460406; doi:10.1007/s11764-024-01580-9)
Supplement: Supplementary file 3 — Supplementary file3 (DOCX 14 KB) [file 11764_2024_1580_MOESM3_ESM.docx]

**Suppl. 6A.** Forest plots of the different scales of the QLQ-C30 questionnaire according to treatment modality. Seminoma served as the reference group. For the functional scales (upper image border), higher scores represent a better outcome, whereas for the symptom scales (lower image border), higher scores represent a worse outcome. QoL= quality of life; *statistically significant p<0.050 after multiple tests.

**Suppl. 6B.** Forest plots of the different scales of the TC module questionnaire according to treatment modality. Seminoma served as a the reference group. For the symptom scales (upper image border), higher scores represent a worse outcome, whereas for the functional scales (lower image border), higher scores represent a better outcome. QoL= quality of life; *statistically significant p<0.050 after multiple tests.
