# Supplementary figures and images for: Long-term quality of life of testicular cancer survivors differs according to applied adjuvant treatment and tumour type
Source: J Cancer Surviv. 2024 Apr 24;19(5):1651–65. doi: 10.1007/s11764-024-01580-9 (PMC12460406; doi:10.1007/s11764-024-01580-9)

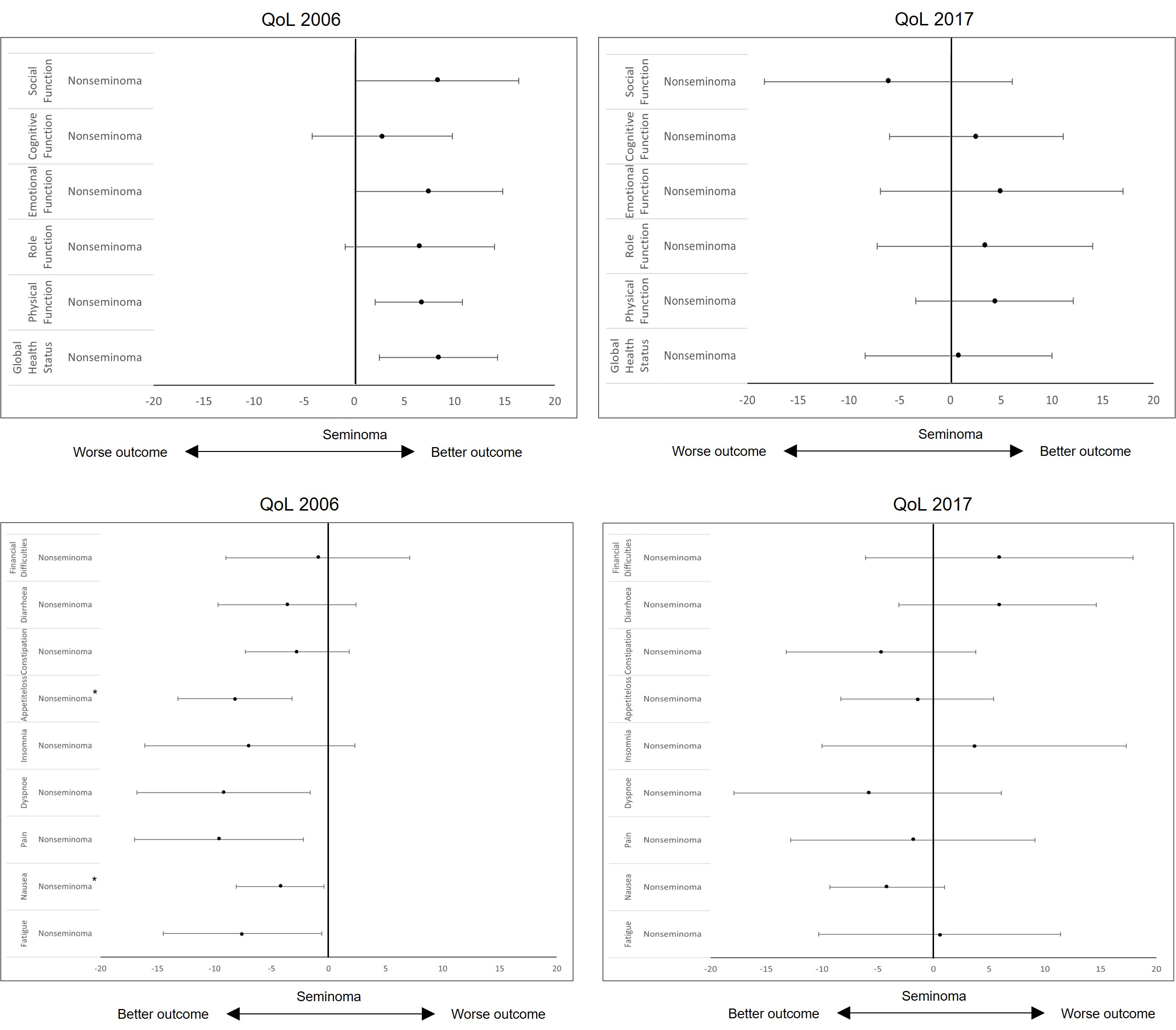

Supplement: Supplementary file 4 — Supplementary file4 (JPG 349 KB) [file 11764_2024_1580_MOESM4_ESM.jpg]

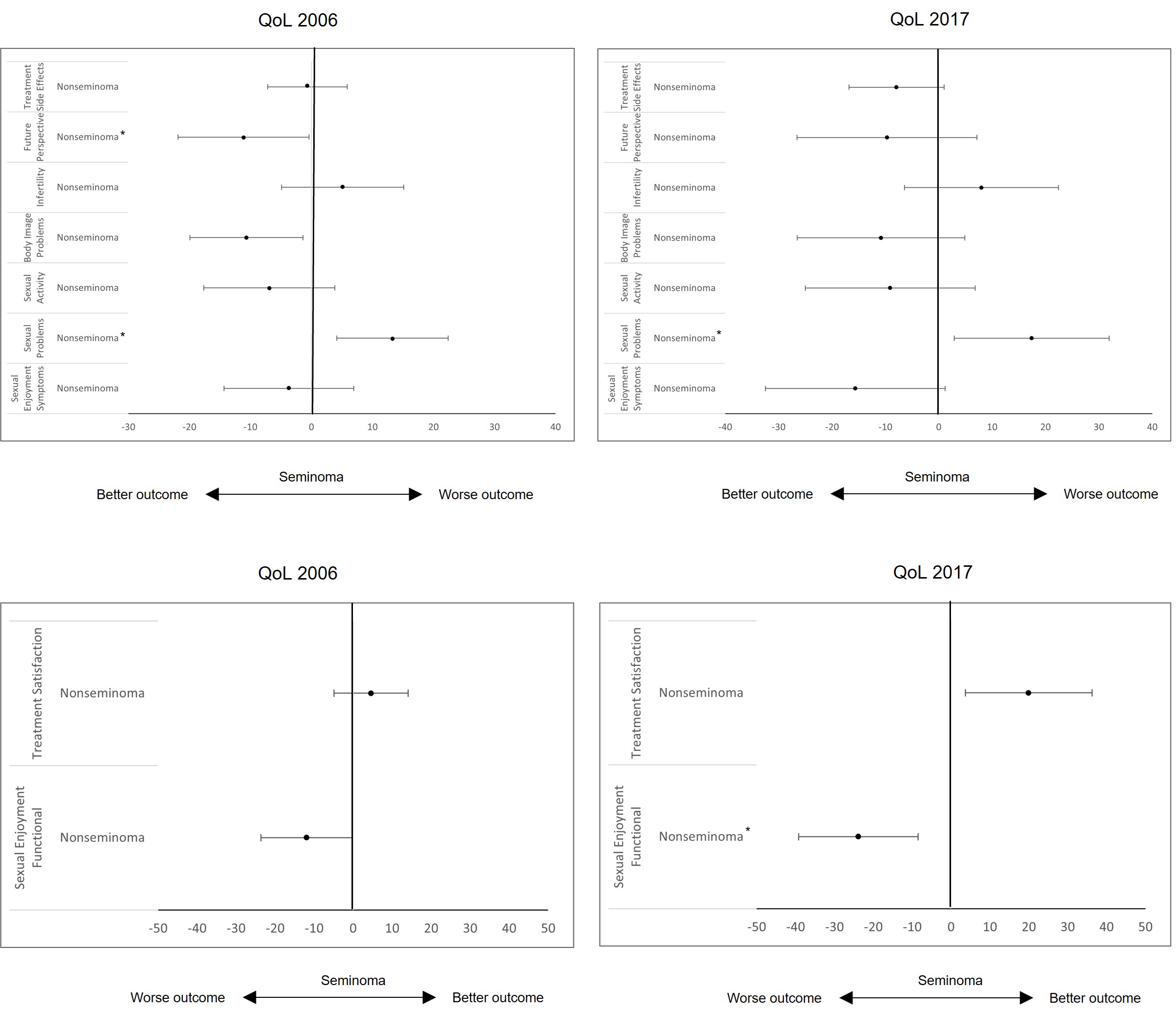

Supplement: Supplementary file 5 — Supplementary file5 (JPG 306 KB) [file 11764_2024_1580_MOESM5_ESM.jpg]
